# The impact of dance activities on the health of persons with Parkinson’s disease in Sweden

**DOI:** 10.1080/17482631.2021.1992842

**Published:** 2021-10-25

**Authors:** Therese Gyrling, Magnus Ljunggren, Staffan Karlsson

**Affiliations:** a School of Health and Welfare, Halmstad University, Halmstad, Sweden; b Faculty of Health Science, Kristianstad University, Kristianstad, Sweden

**Keywords:** Dance, health, parkinson’s disease, qualitative study

## Abstract

**Purpose:** Parkinson’s disease (PD) is associated with motor and non-motor symptoms that negatively influence the person’s quality of life. To reduce illness and increase quality of life, alternative treatments of PD such as dance might be experienced as beneficial. The aim of this study was to explore experiences of how a dance program in Sweden influences perceived physical, social, and emotional wellbeing in persons with PD.

**Method:** A qualitative method with semi-structured interviews and content analysis was used, and 10 participants with variations in age, gender, and how long they had been diagnosed with the disease were interviewed.

**Results:** The results showed that dancing was experienced as improving health, which implied feeling both calmed and excited, getting better sleep, and being able to move with more focus and freedom. The dance program was experienced as a social context through the importance of community, feelings of togetherness, and being able to compare oneself with others. Experiences of self-support included experiencing increased self-esteem and joy, but also a sense of being confirmed and having structure, which gave meaning to everyday life.

**Conclusions:** To reduce illness and increase quality of life in persons with PD, it is important to investigate alternative treatment methods, and this study shows the importance of participating in a dance program in Sweden for the life situation and health of people with PD.

## Introduction

Approximately 18,000–20,000 persons mostly aged over 65 in Sweden are estimated to have Parkinson’s disease (PD) (National Board of Health and Welfare, 2016). PD consists of several of symptoms that have negative impacts on the health of the person with PD. Health can be defined as physical, mental, and social well-being (World Health Organization (WHO), 1948), which may imply subjective feelings of well-being with the availability of positive emotions, joy of life, and social commitment (Ryan & Deci, 2001). The various symptoms from PD affect the physical, mental, and social aspects of the person’s life. PD is associated with motor and non-motor symptoms (Nicoletti et al., 2017) and reduced emotional and cognitive functions, which decreases the persons` quality of life (Frenklach, 2016; Ossowska & Lorenc-Koci, 2013). The motor symptoms increase as more and more neurons in the brain stop functioning, which include hypokinesia, bradykinesia, rigid muscles, and tremors, and the longer the disease progresses, the more speech and facial muscles are affected (Clarke, 2007). Anxiety and depression are the two most common affective symptoms in persons with PD (Carod-Artal et al., 2008; Papapetropoulos et al., 2006). Depression is often an early sign of PD, and thus important to recognize in the pathological pathway of the disease (Lewis et al., 2016). Treatment of depression is primarily focused on drug treatment, but many people find that the medication does not help and side effects are common and lead many people to stop taking the medication (Apeldorf & Alexopoulos, 2003). Having PD implies to handle and cope with several symptoms related to the disease.

Various alternative treatment and intervention are utilized to increase health and wellbeing of persons´ with PD. Persons with PD often have low self-esteem, which impedes their ability to create new social relationships, and they often experience that other people treat them negatively because they often lack facial expressions and have speech disorders (Schwartz & Pell, 2017). A psychological adaptation could be facilitated by support from family members which strengthens the positive approach, while comparing oneself with other persons with PD creates a positive feeling of not being alone and of seeing that there are those who are worse off than oneself (Kang & Ellis-Hill, 2015). As the motor symptoms increase combined with increased cognitive dysfunction, it becomes harder for the person with PD to participate in life and they start to isolate themselves more and more (Schwartz & Pell, 2017). Generally, physical activity is an effective treatment for depression and can in some cases prevent the need for pharmacological treatment altogether (O’Donahue & Cummings, 2011). Persons with PD have a complex morbidity and do not always benefit from pharmacological treatments. Therefore, it is important to investigate other alternative interventions and how persons with PD experience such interventions. Health problems might be favourably impacted by various types of physical activity, which has a good effect on quality of life of persons with PD (Lee et al., 2017). In general, physical activity has been shown to be a good supplement for preventing the development of both somatic and mental illnesses, with studies showing a good effect of increased physical activity on preventing anxiety and depression (Fox, 1999; Pasco et al., 2011; Ströhle, 2009). Physical activity as group training for people aged 65 and over reduces social isolation and contributes, among other things, to reduced depression and reduced drug use (Bath & Gardiner, 2005). Alternative treatment such as physical activity seems to be useful for persons with PD.

Dance can be viewed as being an alternative form of physical activity and exercise for persons with PD. Research have showed evidence that dance can improve motor impairments, specifically balance, and motor symptom severity in persons with mild to moderate PD (Carapellotti et al., 2020). Also, research have studied dance’s effects on non-motor PD symptoms, such as cognitive impairment and depression (Zhang, Hu, Wei, Jia, & Jin, 2019). Dance programs may train a variation of cognitive skills, and previous studies have found that dance can improve spatial cognition (McKee & Hackney, 2013), cognitive switching (Ventura et al., 2016), and mental rotation capabilities (Hashimoto et al., 2015). As dance typically is practiced in a social and enjoyable context, it could decrease isolation and improve psychological benefits. Dance can reduce mood disorders and reduce negative behaviours such as anger (Lewis et al., 2016), and dance activities reduce depression and mental illness in persons with PD while at the same time improving their coordination and balance (Kiepe et al., 2012). Dance has also been shown to improve the quality of life, cognition, and mood of persons with PD (McNeely et al., 2015; Sharp & Hewitt, 2014), and the tango has been shown to have good effects on both balance and well-being (Šumec et al., 2015). Activities such as imitation and mirroring may contribute to motor and non-motor outcomes of dance for people with PD (Bek et al., 2020). There are several of mechanisms through which dance may improve QOL including, among others, improved motor function (Hackney & Earhart, 2009), engagement with music (Hackney & Bennett, 2014), and socialization (Bognar et al., 2017; Holmes & Hackney, 2017).

Previous research on dance for persons with PD has been predominated by quantitative studies with measures of outcomes. Studies with qualitative approaches (e.g., observations, diaries, or interviews) have similarly reported sensorimotor consequences such as improvements in movement quality (Prieto et al., 2021), body awareness, and rigidity in persons with PD participating in ballet (Houston & McGill, 2013) and in the “Dance for PD” program (Westheimer et al., 2015). Another study found that persons with PD experienced that music with a strong rhythmic beat facilitated the management of “freezing of gait” incidents, and hearing footsteps while dancing made them feel more confident and helped them move more easily (Rocha et al., 2017). Participating in a dance class may be experienced by the persons with PD as accepting their own mobility limitations and capabilities and developing a sense of independence in daily life (Heiberger et al., 2011; Houston & McGill, 2013; Prieto et al., 2021). Even though persons with PD identified the treatment of motor symptoms to be the most important goal for dance classes, they also experienced a socialization and relief from feelings of depression and anxiety (Rocha et al., 2017). Dance programs have been shown to provide social interactions and thus decrease isolation and improve quality of life in persons with PD. Persons with PD experienced the dance program as a support group because they felt socially accepted, could self-evaluate their own abilities, and found support for challenges they faced in daily life (Bognar et al., 2017; Prieto et al., 2021). Previous research has shown that dance activities can be an effective complement to traditional drug treatments and that dancing increases motor function and decreases depressive symptoms and social isolation that may result from the symptoms of PD. However, most studies about dance interventions for PD have only investigated measured outcomes, and fewer qualitative studies about the experiences of a dance program in persons with PD have been implemented. To our knowledge no qualitative studies have been performed in Sweden and this study can be a contribution to previous knowledge about experiences of a dance program in persons with PD, also including a Swedish perspective.

## Aim

The aim of the study was to explore experiences of how a dance program in Sweden influences perceived physical, social, and emotional wellbeing in persons with Parkinson’s disease (PD).

## Method design

This was a qualitative study with an inductive approach using semi-structured interviews.

## Intervention

The dance program took place at a dance studio, a place that does not have connotations of rehabilitation or exercise. The dance program was based on the concept of “Dance for Parkinson”. The sessions were held on the same day every week for 12 weeks, and each session lasted 90 minutes and usually had 10–15 participants per session. The dance program was led by a dance leader with a solid background in performing art and who had been educated in the concept of “Dance for Parkinson” (Westheimer et al., 2015). With clear body language, the leader directed and inspired the participants’ movements. The focus was on the experience and not on the workout itself. The session started with everyone imitating the dance leaders’ movements and sounds. In addition to the training of the body, their voices were also activated to correspond to the sound of the music. At one point the participants paired up and mirrored each other’s movements. The participants helped one another, and they participated to the best of their ability, even if this only meant being present for the program. The music was a mix of different styles and beats, including everything from classical to pop music.

## Sample

Interviews were conducted with ten persons who participated in a dance program in the south of Sweden. The inclusion criteria were persons with PD who participated in a dance program once a week, with variation in age, sex, and duration of disease. Of the participating ten persons, there was one man and nine women between the ages of 59 and 81 years. The duration of their diagnosis varied between 4 and 18 years.

## Data collection

The recruitment of participants was done by the dance leader who provided information on the content and purpose of the study to all participants in the dance program who then could choose if they were interested in participating in the study. According to the inclusion criteria, 11 persons were asked by the dance leader if a researcher could get in contact with them and provide further information about the study and the implication for participation. The researchers attended a dance program before the interviews were to begin in order to gain a deeper understanding of the dance program. On-site information was provided on the study, and the potential participants were given the opportunity to ask questions. Thereafter, the time and place of the interview was determined with the persons who chose to participate. Prior to the interviews, written information was given where participants were informed that their participation was voluntary and that they could end their participation at any time during the study. They were also informed that their personal information would be kept confidential and that they cannot be recognized in the material. Written consent was obtained from all participants. The interviews were conducted by the two first authors and were recorded, and all recordings were stored in a locked cabinet. The participants decided where the interviews were to be held, including a site at a hospital, a library, and at the participants’ homes. An interview guide with predetermined questions was used. The questions asked were: “Tell me what dancing means for you”, “What can you tell us about the last time you participated in the dance program (thoughts before, during, and after)”, “What made you start with the dance program”, “If you think about how your life looked before you started dancing, what significance has dancing had for you”, “Can you describe how dancing affects your attitude”, and “How do you see your future regarding dancing”. Follow-up questions were asked as needed for further probing and for clarification (Danielsson, 2017). The interviews lasted between 13 and 31 minutes. After each interview, the content was discussed among the authors to gain a deeper understanding. All interviews were transcribed and encoded.

## Data analysis

A qualitative content analysis according to **Graneheim and Lundman (2004)** was performed where meaning units related to the aim of the study were extracted and then condensed. The condensed meaning units were labelled with codes, and the codes formed sub-categories, which then coalesced into categories. All interviews were read several times by both authors for a deeper and shared understanding as well as for an overall picture of the material. Meaning units were identified and condensed for subsequent coding. The codes were compared based on similarities and differences and then divided into subcategories and categories. The analysis was then validated with the third author (Table I). All codes with similar content formed sub-categories. Categories emerged from the sub-categories that illustrated how persons with PD experienced their health while dancing. Ethical permission was obtained from the local ethical board at Halmstad University (Ref UI 2018/156).

**Table I.** Example of the analysis process

Meaning Unit	Condensed Meaning Unit	Code	Sub-category	Category
It’s not that I think, “I have to drag myself there because I have to”. No. I do it because I want to. I think that it is so fun.	I do it because I want to, not because I have to	To experience meaningfulness	To have something to look forward to	That dancing is experienced as self-supporting
It feels fun and beautiful to make a move that you can do and which you can do as well as possible. There’s a joy in that.	It’s a pleasure to make a move that you actually can manage	To feel joy in activity	To feel	That dancing is experienced as self-supporting

## Results

The content analysis resulted in three categories, which contained nine subcategories (Table II). The category “*Experiencing that dancing improves health*” included how the dance program had positive effects, both physically and mentally. The dance program made the participants feel both calm and excited, and they slept better. It also shifted their focus from their disease and gave them a feeling of liberation. The category “*Being in a social context*” showed the importance of community and feelings of togetherness and of be able to compare oneself with others in a similar situation. The category “*Experiencing dancing as self-supporting*” referred to the overall picture of how the participants experienced how the dance influenced their mental condition. They experienced increased self-esteem and joy, but also a sense of being confirmed and having structure, which gave meaning to everyday life.

**Table II.** The results in categories and sub-categories

Categories	Sub-categories
Experiencing that dancing improves health	To be both calmed and excited To sleep better To move with focus and liberation
Being in a social context	To experience community and togetherness Being able to compare yourself with others
Experiencing dancing as self-supporting	To experience increased self-esteem To feel joy To feel confirmed Getting structure

## Experiencing that dancing improves health

The dance program also affected the participants’ physical factors that in turn contributed to their health. The participants stated that they felt physically better after the dance program, and the effect lasted from a few hours to a few days at a time. The sub-categories included *To be both calmed and excited, To sleep better*, and *To move with focus and liberation*.

### To be both calmed and excited

The participants felt that the dance program gave a sense of peace and quiet and that it was stress-reducing. Some felt that they were lacking physical energy, but the dance program gave them a boost, and they felt more flexible through their ability to coordinate different parts of their body that were not normally used in that way.

“*Well, what to say? It feels relaxing in some way. It feels relaxing*.” *(Interview 10)*

They also felt that they were getting more energy and that they were becoming more spirited. The music was a contributing factor that could feel encouraging.

“*The music is very encouraging … It is very uplifting*.” *(Interview 9)*

### To sleep better

Positive psychological effects were expressed as a better night’s sleep after the dance program.

*“And it’s nice because you sleep better at night too.” (Interview 1)*

### To move with focus and liberation

PD negatively influenced the participants’ health, and the dance program gave a sense of liberation when they had a moment to think and focus on something else besides their disease. They perceived that they could shift their focus from themselves and their illness to instead listening to and following the music and the dance leader. Following the movements and focusing on them instead of their disease gave a sense of liberation and an insight that it was not so hard to move as expected. Thinking about something else also led to increased certainty about the movement pattern.

*“You concentrate on one thing, and you focus on following the music and the movements. Everything else disappears.” (Interview 4)*

*“You are free from the notion that it’s hard to move, because that´s how it feels.” (Interview 5)*

## Being in a social context

Meeting others living with PD created a sense of context for the participants and gave them the feeling that they could be themselves. It was also experienced as an opportunity to jointly find a relationship based on more than just a common disease. The sub-categories were *To experience community and togetherness* and *Being able to compare yourself with others*.

### To experience community and togetherness

The social community was something that permeated all of the interviews. It meant a lot for the participants who felt that the activity had an accepting climate and that it was positive to get out and meet people. Being able to meet others with the same illness meant that they could be themselves and could feel connected with others without thinking about the symptoms of PD as much. The sense of community also made the participants feel empowered to struggle a little more.

*“When you think this was nice, this was fine, and that … Yes, a community. Everyone, that we know each other more or less now, oh, so that, oh, that’s positive.“ (Interview 3)*

*“But here it doesn’t matter. Here we all are … everyone is the same. You can be yourself.“ (Interview 8)*

The community was also a factor that encouraged the participants to attend the dance program every week. They felt that it was important to get away from home and that it felt nice that there was someone who cared and someone who asked how they were.

*“Then it´s very nice to get some stimuli, to follow others. It’s not as fun to sit by yourself and make these moves, it’s the company that does it.” (Interview 1)*

### Being able to compare yourself with others

It was considered a relief to meet others with the same disease in order to be able to compare situations. The participants saw those who were worse off and gained an understanding of their own illness and a sense of gratitude for their own situation. At the same time, there could be a fear of meeting others who were worse off because this might give an indication as to how the disease might affect them in the future. However, this fear disappeared in the meeting with the other participants because it became clear that there were those who had both worse and better situations.

*“Yes, but since I’m so healthy I just got depressed by seeing how I would progress. When I went here to dance, I felt that it was a relief because there are both those who have it worse, who are worse than me, and those who feel better.” (Interview 5)*

*“But it’s good to meet others as I said. Eh, partly because you get an idea of what’s going to happen next … and partly it’s good to get an explanation for other people’s behaviour.” (Interview 9)*

## Experiencing dancing as self-supporting

The participants felt that it was important for them to go to the dance program regardless of their daily condition because the activity gave them more than just physical satisfaction—it created a sense of being needed and of being valued. The sub-categories were *To experience increased self-esteem, To feel joy, To feel confirmed*, and *Getting structured*.

### To experience increased self-esteem

The participants expressed how they had experienced increased self-esteem from the dance program. They felt that they could manage more than they had expected to and that it was a positive feeling to be able to challenge themselves and to be content with their own body. Many participants reported that dancing made a positive change in how they perceived themselves and how they began to think that the disease is not a disease that kills and that it was good to make the most of the situation.

*“Then you’ve thought, ‘I’m doing really well today’. Then you become satisfied with yourself.” (Interview 6)*

### To feel joy

Despite differences in the course of illness and functional level, the dance program contributed to increased health in all participants. Many experienced that it was nice and positive to move to music and that it had a positive impact on their mood and that it was nice to feel that it was possible to participate without the demands for achievement despite various opportunities to perform. The participants also experienced a positive sense of coping with more than they thought they could.

*“And you can see that this is a good thing, that it still has an impact both on the mood … on the mood and physically.” (Interview 5)*

*“But I think the dancing, it’s like a lift and you feel purely euphoric when you leave, in a good mood.” (Interview 4)*

The dance program was described as positive. It provided joy and well-being both before, during, and after the activity, and several participants expressed satisfaction in the feeling they had done something.

*“You feel good when you are there anyway. You look more positive when you leave. More positive in some way.” (Interview 10)*

The combination of dance and music was significant and contributed to prosperity, joy, positivity, and the creation of a community.

*“I feel better about the dance and the music. I do. Because it’s the combination I think, I just think the dance and the movements without music, so I would not have felt the same.” (Interview 4)*

The participants experienced the dance program as meaningful, and it was clear that they looked forward to it because it had positive effects and gave them a sense of satisfaction.

*“Yes, I feel it’s fun to go there. I look forward to it every time.” (Interview 10)*

### To feel confirmed

The participants thought the dance program created an open climate where they felt confirmed by the other participants as well as the dance leader, and they experienced a sense of being seen that contributed to their sense of well-being. It also gave a sense of confirmation that the dance leader knew everyone’s name. To see that a person is participating even while sitting in a wheelchair shows the acceptance and accessibility that the dancing contributed to.

*“He (the dance leader) sees us, we are different, but it feels very positive when I get there, I think.” (Interview 10)*

### Getting structure

The participants felt that it was important to have dance activities once a week in order to create a sense of security in the familiar meeting with other people. They also expressed that the dance program helped them come out and meet like-minded people, and it felt important to have something that made them leave their homes.

*“It’s nice to have something on a regular basis.” (Interview 9)*

## Discussion

We found that people felt physically better after participating in the dance program, and the effect could last for up to several days. Our participants expressed a shift in focus from their illness to instead following the music and the dance leader, which was experienced as a relief and led to feelings of increased safety and improved mobility. This is in line with another qualitative study reporting that persons with PD experienced that music with a rhythmic beat enabled them to manage so-called “freezing of gait” episodes, and hearing footsteps while dancing facilitated them in feeling safer and moving more easily (Rocha et al., 2017). Previous research has also found evidence that the effects of dance can improve movement impairments, specifically balance and motor ability, in persons with PD (Carapellotti et al., 2020). Other studies have shown that engagement with music leads to better motor function in persons with PD (Hackney & Bennett, 2014; Hackney & Earhart, 2009). In this Swedish study, our participants expressed that the dance program gave them a boost of more energy, and they felt more flexible with improved body coordination. Corresponding results were reported in a previous study, where persons in a “Dance for PD” program described increased body awareness and decreased rigidity (Westheimer et al., 2015). For persons with PD, participating in a dance program may be experienced as accepting their own mobility limitations and capabilities, which implies a sense of independence in daily life (Heiberger et al., 2011; Houston & McGill, 2013; Prieto et al., 2021). Evaluation of interventions with dance has showed positive effects on body coordination and balance (Kiepe et al., 2012), and it has been shown that the tango improves balance and well-being in persons with PD (Šumec et al., 2015). Such knowledge may be useful in the recommendation of interventions, including dance, for persons with PD in Sweden.

Participating in a dance program creates a strong sense of community for persons with PD. This Swedish study showed the importance of meeting others with PD and how this gives a feeling of togetherness and at the same time an opportunity to compare oneself with others, as also demonstrated by Kang and Ellis-Hill (2015). The participants with PD in this dance program felt encouraged by the other participants with PD, and this helped them to develop their own resources. The patient leads the way to improvement according to the tide model (Barker & Buchanan-Barker, 2005), and the community that the dance program created gave the participants the opportunity to change. The participants of this dance program experienced that through the dance program they had become part of a community where everyone helped each other with small things, which made it easier in everyday life. Other studies have found that a dance program can be experienced as a support group with social acceptance, self-evaluation of abilities, and support for challenges in daily life in persons with PD (Bognar et al., 2017; Prieto et al., 2021). In this study, the participants were in the same situation and had a desire to come and enjoy the dancing. Previous studies have shown that the absence of social community is associated with increased depressive symptoms (Courtin & Knapp, 2017), while socialization has been found to relieve feelings of depression and anxiety in persons with PD (Rocha et al., 2017). Because dancing, among other things, creates a social community, it can prevent impaired health in persons with PD. Participation in dance plays an important role in everyday life by meeting like-minded people and by reducing cognitive impairment and increasing mobility through physical activity and thus enhancing quality of life (Duncan & Earhart, 2011). The unity between the dancing and the music was experienced by the participants as particularly valuable, and they were able to communicate with others despite various difficulties in expressing themselves with movements, facial expressions, or speech. This study showed that persons with PD experienced well-being by participating in a dance program through togetherness in being part of a community. More knowledge is needed about the importance of social community for persons with PD in order to develop health promotion and alternative treatment methods.

Participating in a Swedish dance program increased self-support in persons with PD. Our results showed that dancing contributed to improved self-esteem, and activation of the body created a positive impact on the emotional difficulties that the disease implied. Improved self-esteem when dancing could be interpreted by the tide model, which argues that life conditions affect mental health and that it is important to use the person’s own resources to implement changes. Supporting the person to use their own resources increases their sense of self-esteem (Barker, 2002). This study showed that joy pervaded this dance program, which is in line with previous studies that have shown that dancing has a positive influence on mood and even has antidepressant effects (McNeely et al., 2015; Sharp & Hewitt, 2014). Dance could also be experienced as a relief of depression and anxiety in persons with PD (Rocha et al., 2017). Dancing as an alternative treatment method provides positive psychosocial effects, and the participants in this Swedish study felt that they had a more self-controlled attitude towards their illness because the dancing allowed them to proactively contribute to their own care. Other studies have shown that participating in a dance program may be experienced as accepting their own limitations and capabilities and developing a sense of independence in daily life in persons with PD (Heiberger et al., 2011; Houston & McGill, 2013; Prieto et al., 2021). Our participants argued that the reason they started the dance program was that many had danced earlier in other forms or had joyful memories related to dancing in their younger years. Other studies have yielded similar results and have shown higher quality of life among those who had danced before they became disabled compared those who had not danced before (Murrock & Graor, 2016), and a sense of being able to perform during a dance program has been shown to give an increased sense of control of the disease and increased self-esteem, to reduce depressive symptoms, and to increase quality of life in persons with PD (Bognar et al., 2017). Because the dancing itself might contribute to increased self-esteem, dancing might be recommended as an alternative treatment method. When introducing a person diagnosed with PD to dance activities, there is a need for a tailored approach to provide increased self-esteem. By motivating and encouraging the person’s own choices, the dance intervention will be health-promoting based on each person’s individual circumstances and will stimulate a sense of meaningfulness and independence.

## Methodological considerations

A qualitative method was used because such a method focuses on interpretations of the text and is commonly used in healthcare research (Lundman & Hällgren-Graneheim, 2012). Because the participants’ experiences were to be investigated, an inductive approach was used, with an analysis of the text based on the participants’ stories and experiences. This provides good validity for the study. A weakness of the method could be that the participants might have given answers that they thought the interviewer would want to hear (Kjellström, 2017). The participants knew in advance that the study was about health, which may have resulted in the answers focusing mostly on the positive effects of the dance program. The dance leader chose which participants would be included in the study based on the inclusion criteria, and this meant that even participants with difficulty expressing themselves were included, which contributed to an increased variety in disease progression among the participants. Of the persons who matched the inclusion criteria, only one chose not to participate in the study. The participants mostly consisted of women, and thus the results reflect women’s experiences more than men’s. Prior to the interviews, the researchers visited the dance program where participants received information about the study and were given the opportunity to ask questions. This was also done to gain a deeper understanding of what the dance program contained. In addition, the hope was that the participants would feel more comfortable when they met the researchers during the interviews. The two first authors performed the interviews, where one acted as the observer and the other conducted the interview. The roles were switched between each interview. With the two first authors participating in the interviews, a discussion could be initiated afterwards and follow-up questions as well as approaches could be adjusted. Having two researchers at the interviews might have made the participant feel uncomfortable, and this was attempted to be addressed by the researchers meeting the participants previously and thus contributing to a relaxed environment and good conversation climate.

Some interviews were short, which may have been influenced by the symptom from PD and its effects on cognitive functions. The participants repeatedly expressed difficulties in articulating themselves and in responding to questions about their emotions and mood. They also expressed difficulty in understanding the questions, and the questions could therefore have been reformed with more focus on emotions and experiences. However, a final question was asked to catch if there was anything that the participant experienced that we did not cover or that they would like to add. The participants had varied disease symptoms, which meant that there were those who wanted to bring somebody with them to the interviews, either as support or to clarify what was said. The choice to include the participants who needed some kind of assistance was made due to the fact that it felt important to include their opinions as well. The data processing took place by the two first authors and was validated with the third author to ensure the quality of the study and to gain a deeper understanding of the content.

## Conclusion and implication

This study showed that persons with PD experienced improved health by participating in the Swedish dance program. The dance program had positive physical and psychosocial effects through an increased sense of togetherness and community with the other participants. The participants also experienced increased self-esteem and happiness by having the chance to activate their body, and this contributed to their improved mood. The results regarding the impact of dancing on the health of persons with PD might lead to better allocation of resources in order to offer alternative treatment methods for health promotion purposes. The results of this study and similar studies need to reach the healthcare professions through education and information in order to offer persons with PD the opportunity to participate in dance activities in Sweden and thus be able to share in the positive effects that such activities provide. It is important to further investigate alternative treatment methods in PD to increase health and quality of life.
